# Heart–brain interaction in cardiogenic dementia: pathophysiology and therapeutic potential

**DOI:** 10.3389/fcvm.2024.1304864

**Published:** 2024-01-24

**Authors:** Jiaxu Liu, Guangxu Xiao, Yujuan Liang, Shuang He, Ming Lyu, Yan Zhu

**Affiliations:** ^1^ State Key Laboratory of Component-Based Chinese Medicine, Tianjin University of Traditional Chinese Medicine, Tianjin, China; ^2^ Tianjin Key Laboratory of Translational Research of TCM Prescription and Syndrome, First Teaching Hospital of Tianjin University of Traditional Chinese Medicine, Tianjin, China

**Keywords:** cardiogenic dementia, cognitive impairment, heart–brain interaction, heart–brain axis, heart disease

## Abstract

Diagnosis and treatment of patients with cardiovascular and neurologic diseases primarily focus on the heart and brain, respectively. An increasing number of preclinical and clinical studies have confirmed a causal relationship between heart and brain diseases. Cardiogenic dementia is a cognitive impairment caused by heart dysfunction and has received increasing research attention. The prevention and treatment of cardiogenic dementia are essential to improve the quality of life, particularly in the elderly and aging population. This study describes the changes in cognitive function associated with coronary artery disease, myocardial infarction, heart failure, atrial fibrillation and heart valve disease. An updated understanding of the two known pathogenic mechanisms of cardiogenic dementia is presented and discussed. One is a cascade of events caused by cerebral hypoperfusion due to long-term reduction of cardiac output after heart disease, and the other is cognitive impairment regardless of the changes in cerebral blood flow after cardiac injury. Furthermore, potential medications for the prevention and treatment of cardiogenic dementia are reviewed, with particular attention to multicomponent herbal medicines.

## 1 Introduction

Heart failure (HF) is the fastest-growing cardiovascular disease and a major threat to global public health (1). Dysfunctions of other organ systems due to decreased heart function can reduce the quality of life of patients and increase their treatment costs and mortality (2). In addition, epidemiological and clinicopathological studies have reported a causal relationship between heart disease and dementia, which is expected to significantly increase the risk of developing dementia among patients with heart disease (3). Consequently, these conditions place a significant burden on the public and healthcare systems worldwide.

The term “cardiogenic dementia” was coined as early as 1977 and was defined in 1982 as a cardiocerebral syndrome with cognitive impairment after heart disease (4, 5). After a few decades of intense research, coronary artery disease (CAD), myocardial infarction (MI), HF, atrial fibrillation (AF), and valvular heart disease (VHD) are the primary risk factors for cardiogenic dementia. To date, two potential disease mechanisms have been identified. (1) Heart disease causes chronic cerebral hypoperfusion (CCH) by reducing cardiac output. CCH, in turn, induces oxidative stress, inflammatory response, immune response, blood–brain barrier (BBB) damage, and amyloid-beta protein (Aβ) deposition, resulting in brain tissue damage, cognitive impairment, and even dementia. (2) In patients with heart disease who do not suffer from CCH, myocardial damage directly damages cognitive function through systemic inflammation and neurohumoral activation. These findings demonstrate that heart rate variation can activate the posterior insular cortex to encode fear and anxiety behaviors. Therefore, this study reviews and analyzes the causes, clinical consequences, and prevention and treatment strategies of heart disease-induced dementia. In this study, we revealed the risk factors underlying the incidence of cardiogenic dementia. The summarized pathophysiological mechanisms of cardiogenic dementia provide a new perspective for drug development. A better understanding of heart–brain interactions is expected to facilitate the development of evidence-based treatments for elderly patients.

## 2 Risk factors of cardiogenic dementia

Because the brain is a high-energy-consuming organ, a long-term decrease in cardiac output leads to CCH, which causes structural changes and partial dysfunction (6). Therefore, heart diseases characterized by low cardiac output, such as CAD, MI, HF, AF, and VHD, are major risk factors for cardiogenic dementia. On the other hand, the complex pathogenesis of these heart diseases makes them risk factors for cardiogenic dementia independent of cerebral hypoperfusion as discussed below.

### 2.1 AF

As a prevalent cause of cognitive impairment, AF is also a risk factor for cardiogenic dementia. AF may increase the risk of cognitive impairment or dementia by increasing the risk of stroke (7, 8). However, a community-based longitudinal cohort study revealed that even in the absence of stroke, individuals diagnosed with AF may develop cognitive impairment or dementia at an earlier age compared with those without a history of AF (9, 10). These findings indicate that AF events independently increase the risk of dementia, possibly due to cardiogenic thrombosis caused by AF. Cardiogenic thrombi formed by AF can enter the blood circulation and block cerebral vessels, leading to vascular dementia and ischemic stroke (11). In addition to promoting thrombosis by altering cardiac hemodynamics, AF may also cause platelet activation, which in turn promotes Aβ fibril aggregation, thereby triggering cognitive impairment (11, 12). This may also be an important reason why many retrospective observational and prospective studies suggest that anticoagulants improve cognitive impairment in patients with AF (13). What cannot be ignored is the impact of cerebral hypoperfusion caused by AF on cognitive function. The decreased stroke volume due to beat-to-beat variation in AF is an important cause of CCH. A large cohort from the AGES-Reykjavik study involving 2,291 participants (average age of 79.5 years) found that the average total cerebral blood flow (CBF) of the persistent AF group (systolic blood pressure 141.3 ± 22.8 mm Hg) was significantly reduced compared with that of the paroxysmal AF group (systolic blood pressure 142.7 ± 22.6 mm Hg) and the non-AF group (systolic blood pressure 145.3 ± 20.7 mm Hg) (14). In addition, it was found that AF induced high variability in cerebral hemodynamic variables in lumped-parameter models simulating AF, leading to severe cerebral hemodynamic events such as arteriolar hyperpressure and reduced blood flow (15). Cerebral hypoperfusion caused by AF triggers a series of events that reduce cognitive function, such as vascular inflammation, brain atrophy, and cerebral white matter lesions (16, 17). Therefore, anticoagulation and rhythm/heart rate control against AF may be an effective approach to reduce the risk of cardiogenic dementia.

### 2.2 CAD

Notably, a positive correlation was observed between CAD and cognitive decline including dementia. However, treating CAD alone does not significantly suppress the rapid increase in the number of individuals at risk of dementia, which may be due to the increased risk of cardiogenic dementia (18). CAD can easily lead to cardiac ischemia and decrease myocardial contractility, which makes it difficult to maintain sufficient cerebral perfusion. This is an important reason for inducing cardiogenic dementia. Magnetic resonance perfusion imaging study found that compared with the healthy control group (age 59 ± 8 years, systolic blood pressure 120.28 ± 15.97 mm Hg, heart rate 58.34 ± 9.54, ejection fraction 67.41 ± 9.82%), CAD patients (age 59 ± 6 years, systolic blood pressure 126.70 ± 21.20 mm Hg, heart rate 59.11 ± 6.85 beat/min) had reduced CBF and cerebrovascular reactivity in multiple brain regions, including the anterior cingulate, insula, postcentral, and superior frontal regions (19). Moreover, a cross-sectional study involving 673 CAD patients found that low Montreal Cognitive Assessment (MoCA) scores in CAD patients (age 66.3 ± 6.5 years, 24% of females, systolic blood pressure 140.5 ± 27 mm Hg) were associated with a lower level of brain-derived neurotrophic factor (BDNF) released by platelets in the serum (20). The level of BDNF, a neuronutrient that prevents cognitive decline, is negatively correlated with the occurrence of Alzheimer's disease (AD) (21, 22). In another cross-sectional study of 129 CAD patients, it was found that CAD patients with mild cognitive impairment had significantly lower serum levels of dihydro-gamma-linolenic acid (DGLA) than those with normal cognitive function, indicating that DGLA may be an important marker for identifying early cognitive decline in CAD patients (23).

### 2.3 MI

Although the effect of MI on dementia is still somewhat debatable, recent clinical and preclinical studies have confirmed that cognitive impairment caused by MI plays a significant role in the development of cardiogenic dementia. A multicenter registration study revealed that 29.8% of MI patients (age 73.1 ± 6.5 years, 37.8% females, 77.4% with hypertension) developed outbound mild cognitive impairment, whereas 25.8% (age 73.9 ± 6.6 years, 49.7% females, 81.9% with hypertension) had moderate/severe cognitive impairment (24). Elsewhere, a Rotterdam Scan Study found that patients with MI had increased white matter lesion load and the probability of cerebral infarction, especially those with undiagnosed MI (25). These results may be mainly attributed to CCH caused by MI. Patients with MI often have irreversible myocardial fibrosis and systolic dysfunction, resulting in reduced cardiac output (26, 27). Even when systolic blood pressure is maintained relatively well, patients with MI may develop peripheral hypoperfusion, which may be related to vasoconstriction (28). Therefore, these pathways are crucial for the occurrence of CCH after MI. In addition to CCH, the systemic inflammatory response caused by MI is an important factor contributing to cardiogenic dementia. MI-induced release of inflammatory factors may lead to BBB disruption and then trigger neuroinflammation, which is a precursor of neurodegeneration (29–31). Of note, patients with MI have a low risk of AD but a high risk of vascular dementia, which may be attributed to the cerebral small vessel disease caused by MI (32).

### 2.4 HF

Cognitive impairment has been frequently described as a consequence of HF in the past decades. A meta-analysis showed that HF is associated with a 60% increase in the risk of dementia (33). Low cardiac output of HF patients is one of the key reasons for cerebral hypoperfusion, which is critical to cognitive function. Another cause of CCH is the impairment of vascular autoregulation after HF (34, 35). Disruption of vascular autoregulation impairs vasodilation function, making it difficult to maintain sufficient CBF in a state of low cardiac output. Additionally, systemic inflammation caused by HF is also an important factor leading to cognitive impairment. A cross-sectional study of 270 HF patients found that baseline smoking (*χ*2 = 6.33), unmarried (*χ*2 = 12.0), hypertension (*χ*2 = 5.72), higher body mass index (*d* = 0.45), and physical fatigue (*d* = 0.25) were associated with higher C-reactive protein (CRP) levels. Cross-sectionally, CRP levels were negatively correlated with MoCA scores (36). The presence of these conditions makes HF an important cause of cardiogenic dementia. In addition to HF with reduced ejection fraction (HFrEF), HF with preserved ejection fraction (HFpEF) also exhibits cognitive impairment (37). A population-based Rotterdam study also found that poor diastolic function also increased the risk of dementia (38). Age and gender are also contributing factors to the risk of HF-induced dementia. A cohort study from the Danish Medical Registry discovered a higher risk of dementia among men and HF patients aged below 70 years (39).

### 2.5 VHD

VHD is an important risk factor that causes cardiogenic dementia, either by inducing HF and AF or by itself causing cerebral hypoperfusion. Several clinical studies have shown that VHD increases the risk of cognitive impairment and even stroke (40). Aortic stenosis is the most common in VHD, and a decrease in valve area increases the left ventricular pressure load. Although the development of myocardial hypertrophy can compensate for this pressure overload to maintain a normal ejection fraction, systolic function begins to decline when the limit is exceeded (41). Normal cardiac systolic function is the key to maintaining cerebral perfusion. In mitral regurgitation caused by venous drainage, CBF is decreased and brain metabolism is imbalanced (42). A prospective study also showed that patients with mitral regurgitation (age 71.1 ± 12.2, 25% women, 55% with hypertension) had a higher risk of developing cognitive impairment than controls (age 71.1 ± 12.2, 25% women, 37.5% with hypertension) (42). Notably, oxyhemoglobin increases during exercise were significantly lower in VHD patients than those in normal individuals, even with normal resting cardiac output (43). This suggests that VHD patients have cerebral hypoperfusion during exercise.

## 3 Potential mechanisms of cardiogenic dementia

**Figure 1:**
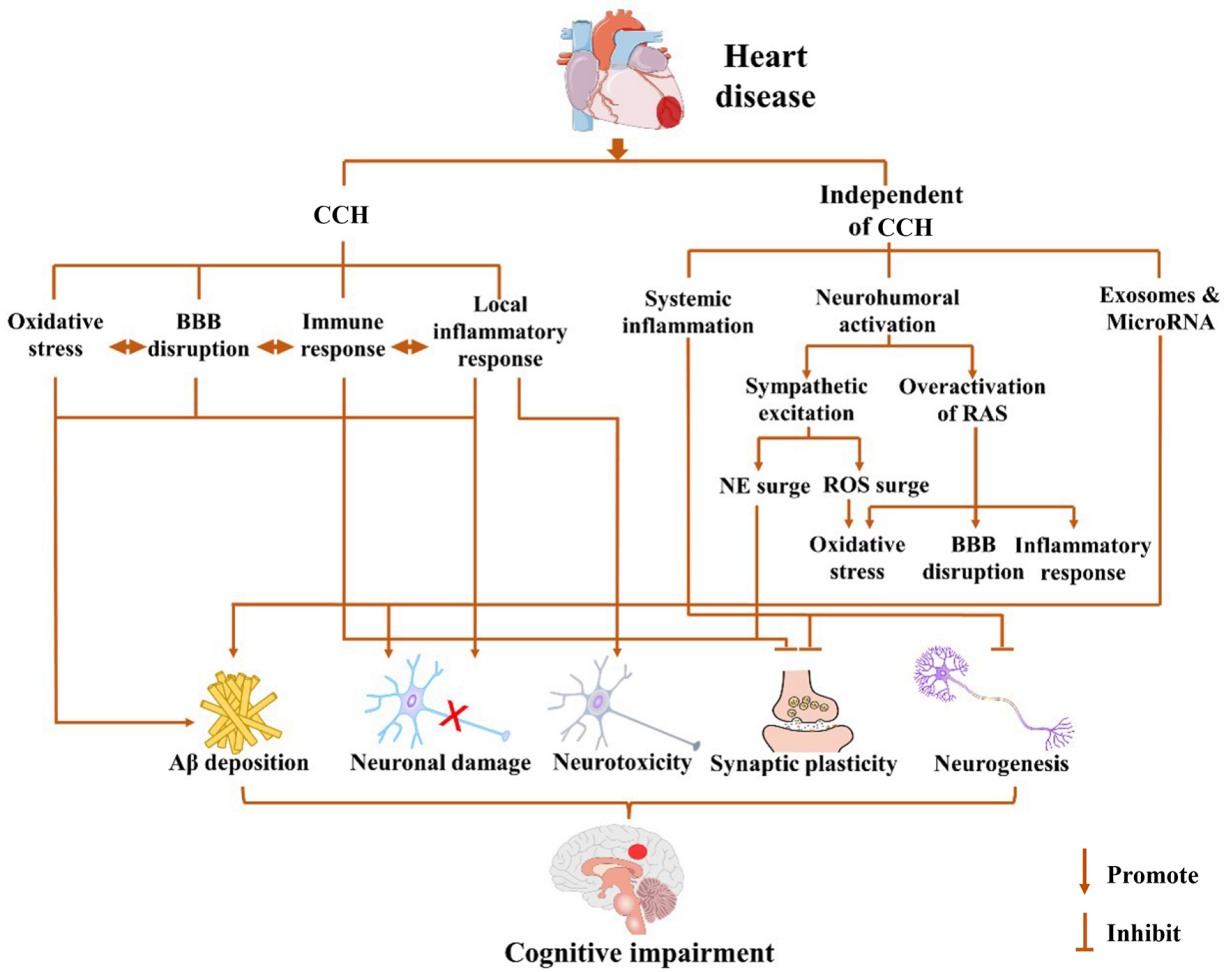
Possible mechanisms for cardiogenic dementia. Cognitive impairment may occur after pathological cardiovascular events in a chronic cerebral hypoperfusion (CCH)-dependent or -independent way. Long-term decline of cardiac output after heart disease leads to CCH, which triggers a cascade of events including oxidative stress, local inflammatory response, immune response, and blood–brain barrier (BBB) disruption. Cardiogenic dementia may occur independent of cerebral blood flow (CBF) changes after cardiac injury, which involves systemic inflammation, neurohumoral activation, and exosome release. The sympathetic excitation may cause the increase of norepinephrine (NE) and reactive oxygen species (ROS), while the overactivation of the renin–angiotensin system (RAS) also leads to oxidative stress, BBB disruption, and inflammation response. In summary, heart disease promotes amyloid-beta protein (Aβ) deposition, neuronal damage, and neurotoxicity and inhibits synaptic plasticity and neurogenesis through CCH-dependent or CCH-independent ways. The interaction among them further exacerbates the damage to cognitive function.

Heart disease may cause cardiogenic dementia through various mechanisms primarily categorized into two types: a series of events caused by CCH caused by the long-term reduction of cardiac output after heart disease and the underlying mechanism of cognitive impairment due to cardiac injury, which is independent of changes in CBF. These two mechanisms are involved in the development of cardiogenic dementia. The mechanisms underlying cognitive impairment after heart disease are summarized in Figure 1.

### 3.1 Potential mechanisms of cognitive impairment through cerebral hypoperfusion after heart disease

CCH after heart disease is one of the main mechanisms underlying the development of cognitive impairment. While ischemia/hypoxia dilates blood vessels to maintain adequate CBF through blood gas regulation, cerebral autoregulation, and neurovascular coupling (NVC), these modulations fail in patients with heart disease, especially at later stages (44, 45). Although elevated levels of CO_2_ in the blood after CCH relax cerebral arterioles via reducing vascular smooth muscle tension to increase CBF, this compensatory mechanism is limited (46, 47). In a study of 50 patients with different degrees of HF, it was found that their cerebrovascular reactivity is impaired, which leads to the inability of CO_2_ to further relax the cerebrovasculature (48). Furthermore, in a cerebral hemodynamics study of 23 HFrEF patients, 8 HFrecEF patients, and 13 healthy controls, the transcranial Doppler results indicate that the NVC of HFrEF patients is significantly impaired, and visual stimulation-induced increase in CBF velocity is decreased (49). In addition, neurohumoral activation, one of the hallmarks of HF, also plays a key role in regulating vascular tension. Abnormally elevated vasopressin secretion and activation of the renin–angiotensin system and sympathetic nerve activity have been reported in dog and rat models of HF (50). These increase the tension of peripheral blood vessels (50) and also limit the further relaxation of cerebral vessels. As far as AF patients are concerned, the NVC responses to visual stimulation were also blunted compared to age-matched healthy controls (51). At the same time, a magnetic resonance imaging scan showed a decrease in the total gray matter volume of AF patients (52), and this subclinical neurodegeneration may be one of the reasons for the reduction of NVC in patients with AF. In conclusion, patients with heart disease develop CCH in a state of low cardiac output due to changes in cerebrovascular reactivity to CO_2_, cerebral autoregulation, and NVC. A series of CCH-induced events that impair cognitive function are discussed below.

#### 3.1.1 Oxidative stress

Due to cerebral hypoperfusion caused by decreased cardiac output, mitochondrial dysfunction produces enormous reactive oxygen species (ROS) and oxidative stress (53, 54). In addition, a study using bilateral common carotid artery occlusion to establish CCH models in rats also found that CCH caused mitochondrial damage and a large amount of ROS production in microglia (55). Excessive activation of oxidative stress causes damage to hippocampal neurons, which leads to cognitive dysfunction (56). Meanwhile, in a rat model of sucrose-induced metabolic syndrome (MetS), activated oxidative stress was found to promote the synthesis of amyloid precursor protein (APP) and Aβ by increasing the activity of β-site APP cleaving enzyme 1 (BACE-1) (57). Notably, abnormal Aβ accumulation and hyperphosphorylated tau promote redox imbalance (58). In a mouse model of Aβ-induced AD, the level of ROS in the brain tissue of mice was significantly increased after intraventricular injection of Aβ_1−42_, and oxidative stress was aggravated (59). These events create a vicious circle that promotes the occurrence and development of cognitive impairment.

#### 3.1.2 Local inflammatory response

Inflammation caused by cardiac disease may interact with oxidative stress, leading to the worsening of cognitive impairment. Heart disease causes local and systemic inflammation in the brain via CCH. High-mobility group box protein 1 (HMGB1) and pro-inflammatory cytokines such as tumor necrosis factor-α (TNF-α) and interleukin-1β (IL-1β) in the cortex and hippocampus of CCH mice induced by bilateral carotid artery stenosis (BCAS) have been reported to significantly increase (60). A clinical study found that the concentration of IL-1β in the serum of HF patients was higher than that of the non-HF and control groups (61). IL-1β, a pro-inflammatory cytokine, is an important factor driving inflammatory responses in the central nervous system (62, 63). The increase in IL-1β and the resulting inflammatory process disrupt cognitive function (62, 63). Furthermore, abnormal elevation of IL-1β was found to affect the consolidation of hippocampus-dependent memory by administering IL-1β in the dorsal hippocampus of rats (63). Cognitive function impairment attributed to IL-1β may also be linked to reduced glutamate and brain-derived neuronal factor (BDNF) release and increased p38 and ERK phosphorylation in the hippocampus (63). In addition, TNF-α promotes the occurrence of inflammatory response and causes brain damage through neurotoxicity (64). During CCH in BCAS mice, the activated ASK1-p38-TNF-α pathway disrupts the integrity of the BBB, causing IL-6 production (65). A series of protective mechanisms in the brain are also stimulated during CCH. For example, increased CD73 after CCH can downregulate IL-1β, IL-6, and TNF-α expression levels in brain tissues of BCAS mice by releasing adenosine, thereby exerting protective effects (66).

#### 3.1.3 Immune response

Accumulating evidence supports the involvement of CCH-induced immune responses in the synthesis and deposition of Aβ, hence promoting cardiogenic dementia progression (67, 68). A retrospective study involving 196 AF patients and 47 non-AF control patients found that astrocyte-specific glial acidic fibrillary protein (GFAP) and microtubule-associated tau protein were substantially elevated in the serum of AF patients (69). These results indicate that astrocytes significantly proliferate during AF. In addition, a precise 3D morphometric analysis of single microglia and astrocytes in specific brain nuclei of HF rats using a microglia/astrocyte analyzer revealed that microglia and astrocytes in the central amygdala had undergone structural remodeling and transition to the pro-inflammatory phenotype (70). The significant proliferation of activated microglia and astrocytes in the brain was observed not only in animal models of HF but also in animal models of BCAS-induced CCH, suggesting that CCH is one of the important causes of immune responses in the brain (71). Activated microglia show impaired Aβ clearance due to the disrupted phenotype, which further increases Aβ accumulation (72). Overactivated microglia participate in the phagocytosis of synapses to mediate synaptic loss and aggravate the tau pathological process (72). Activated microglia were also found to aggravate tau pathology and memory impairment in the hTau mouse model (73). Neuroinflammation caused by active astrocytes in the brain tissue of MI mice is also an important process that aggravates cognitive impairment (74).

#### 3.1.4 BBB disruption

In the case of CCH induced by reduced cardiac output, the blood flow regulation mechanisms in the brain are unable to sustain normal metabolism of brain tissue in the long term, also disrupting the BBB (75, 76). In the animal model of BCAS, CCH can induce pericytes to separate from their original positions in blood vessels, thereby increasing the permeability of the BBB through endothelial endocytosis (77, 78). This direct damage to the BBB may precede a local inflammatory response (77, 78). In a mouse model of AD with chronic hypoperfusion-transgenic mice (PS1V97l) with right common carotid artery ligation, CCH significantly upregulates the receptor for advanced glycation end-products (RAGE) on the BBB of AD mice, which in turn promotes the accumulation of Aβ (79). Meanwhile, RAGE also enhances oxidative stress and inflammation by activating the NF-κB pathway, further accelerating the deletion of tight junction proteins (79). Additionally, the oxidative stress, local inflammatory, and immune responses mentioned above also exacerbate the breaking of the BBB. The decomposition of the BBB results in the obstruction of Aβ clearance through endocytosis and pathological secondary effects, such as the accumulation of harmful substances, leukocyte infiltration, and white matter damage, which are mostly prevalent in vascular dementia.

### 3.2 Potential mechanisms of cognitive impairment independent of changes in CBF after heart disease

The cerebral hypoperfusion caused by heart disease is a crucial mechanism in the pathogenesis of cardiogenic dementia. Other studies have shown that patients with heart disease develop cognitive impairment and even dementia even though CBF is not significantly impaired (80). The pathological mechanisms of this condition are discussed below.

#### 3.2.1 Systemic inflammation

Systemic inflammation triggered by heart disease may lead to cognitive impairment, in conjunction or together with CCH. The inflammatory response caused by heart disease is not limited to the myocardial tissue, and a large number of pro-inflammatory factors including IL-6, TNF-α, IL-1β, and monocyte chemoattractant protein-1 (MCP-1) also reach the brain through blood circulation. The concentrations of IL-6, TNF-α, IL-1β, and MCP-1 not only evidently increased in the myocardial tissue of mice with acute MI established by ligation of the left anterior descending artery (LAD) but also significantly elevated in serum (81, 82). These inflammatory factors disrupt the integrity of the BBB, leading to neuroinflammation, which in turn exacerbates cognitive impairment (83). Additionally, IL-6, TNF-α, IL-1β, and other pro-inflammatory factors participate in the alteration of complex cognitive processes, such as synaptic plasticity, neurogenesis, and neuromodulation, and directly suppress the cascade of neurotransmitters related to learning and memory (84).

#### 3.2.2 Neurohumoral activation

Neurohumoral activation is a mechanism of cardiogenic dementia independent of cerebral hypoperfusion, including sympathetic excitation and overactivation of the RAS (RAS). It was found that cardiac norepinephrine (NE) spillover in mild to moderate congestive heart failure (CHF) patients was three times higher than that in healthy subjects, while cardiac NE spillover in the severe CHF group was four times higher by using the radioactive tracer method (85). HF-induced neurohumoral activation has been reported to downregulate synaptic plasticity of hippocampal neurons in HF rats (86). Studies have shown that cognitive dysfunction in HF rats is related to the inhibition of the Wnt/β-catenin signaling pathway in hippocampal neurons (80). This may be due to the internalization of β-adrenergic receptors (β-ARs) by a large amount of NE that reduces the expression of cAMP and protein kinase A (PKA), thereby inhibiting the activity of the canonical Wnt pathway (87–89). In addition, the Wnt/β-catenin signaling pathway is implicated in synaptic assembly, neurotransmission, and regulation of synaptic plasticity, and thus inhibition of this pathway aggravates cognitive impairment (80, 90). In addition to reducing CBF, hyperactivated RAS exacerbates inflammatory responses and oxidative stress in the brain and disrupts the integrity of the BBB (91, 92).

#### 3.2.3 Extracellular vesicles (EVs) and microRNA (miR)

Although the current understanding of the mechanisms of heart–brain interaction is still limited, emerging evidence suggests that EVs and their cargo miRs mediate organ-to-organ or cell-to-cell communication (93). The significantly increased EVs in the plasma of patients with heart disease may carry IL-1β and TNF-α and other pro-inflammatory cytokines transmit “danger or inflammatory signals” to other organs or cells, especially cardiomyocyte-derived EVs (94, 95). The number of EVs passing through the BBB increases by several orders of magnitude after MI, and encapsulated pro-inflammatory factors may be transmitted to brain tissue through endocytosis in the brain (96). However, these may be absorbed by astrocytes and microglia to trigger neuroinflammation, further impairing cognitive function (97). Therefore, EVs are considered important mediators of heart–brain interactions. Furthermore, miR-1, miR-133, mir-208a, and miR-499, which are considered to be cardiac-specific, were also clearly altered in the plasma of MI patients (98). Previous studies indicate that exosomes transport cardiac-derived miR-1 into hippocampal neurons following MI and disrupt the microtubule structure of mouse hippocampal neurons by suppressing the expression of TPPP/p25 protein (99). After miR-1 overexpression caused by MI, exosomes could transport miR-1 into the hippocampus of the brain and attenuate exocytosis of synaptic vesicles in the hippocampus of mice by reducing SNAP-25 protein expression (100). Notably, the reduction of β-catenin in the Wnt/β-catenin pathway associated with cognitive dysfunction of HF rats may be partly attributable to cardiac-derived miRs after HF (80, 101, 102).

## 4 Potential drugs for the prevention and treatment of cardiogenic dementia

In view of the increasing incidence of cardiogenic dementia, there is an urgent need to identify medications that can treat heart disease while preserving cognitive function. Recent clinical studies showed that commonly used statins, antithrombotic drugs, and some traditional Chinese medicine (TCM) for heart disease are capable of improving cognitive impairment while exerting their cardiovascular effects.

### 4.1 Statins

Statins are known to be effective in reducing the morbidity and mortality of cardiovascular and cerebrovascular diseases. At the same time, the efficacy of statins on the cognitive function of patients with heart disease has received extensive research attention. A nationwide retrospective cohort study discovered that the application of statin is beneficial in reducing the risk of dementia in patients with ischemic heart disease, with rosuvastatin having the most predominant preventive effect of dementia (103). Rosuvastatin may exert anti-inflammatory effects by inhibiting the nuclear factor kappa-B (NF- κB) signaling pathway, thereby reducing the risk of cognitive impairment (104). Interestingly, analysis of high-quality randomized controlled trials and prospective cohort studies indicated that statins do not affect cognition in the short term but have a positive role in dementia prevention in the long term (105). However, large-scale longitudinal cohort studies are still needed to further establish the role of statins in cardiogenic dementia.

### 4.2 Anticoagulants

Among the antithrombotic drugs, findings on oral anticoagulants and the risk of dementia in patients with heart disease are largely established. Evidence from clinical studies suggests that direct oral anticoagulants (DOACs) reduce the risk of dementia in AF patients, and edoxaban, a member of DOACs, is associated with a lower risk of developing dementia in AF patients compared to warfarin (106). In contrast to vitamin K antagonists, patients receiving DOACs have a lower risk of dementia or mild cognitive impairment (107). Studies have shown that Aβ released into the blood promotes platelet aggregation and catalyzes the conversion of fibrinogen to fibrin by activation of thrombin, resulting in degradation-resistant, Aβ-containing fibrin clots. These clots, together with oligomeric Aβ, trigger vasoconstriction and cerebral amyloid angiopathy, whereas DOAC exerts an antithrombotic effect by targeting pathological thrombin to reduce the risk of cognitive impairment (108). Moreover, the treatment effect of DOACs is associated with patient age and stroke history (106). For example, cilostazol reduces the risk of dementia in patients with ischemic heart disease and is more effective for the prevention of dementia in female patients (109).

### 4.3 Traditional Chinese medicine

Clinical studies have shown that single-molecule drugs with a specific target are less effective in the prevention and treatment of complex diseases such as stroke and heart-related brain diseases. Herbal medicine, such as TCM, offers an attractive alternative because of its multiple-component nature. Many TCM formulas have demonstrated the capability of regulating the crosstalk between different pathways through multiple targets, thereby simultaneously reducing inflammation, inhibiting oxidative stress damage, regulating autophagy, and ultimately preventing and treating dementia (110).

Garlic is often used as an important adjuvant in TCM. According to the TCM theory, it has the effect of “fortifying the spleen and harmonizing the stomach” (111). Modern pharmacological studies have shown that aged garlic extract (AGE) reduces the risk of heart disease and dementia because of its multiple pharmacological effects including antioxidative stress, anti-inflammation, and lipid metabolism regulation (111). A clinical study of 93 arteriosclerosis patients aged 40–75 years found that AGE may effectively increase peripheral tissue perfusion and microcirculation by increasing NO release and improving endothelial function after 12 months of treatment (112). Moreover, after administration to mice, AGE increased NO production by 30%–40% via activation of constitutive NO synthase (cNOS), which was conducive to further vasodilation (113). Therefore, AGE may improve cardiac function while maintaining the CBF required for brain activity by further dilating the cerebral arterioles. In addition, a preclinical study found that 2-month treatment with AGE mitigated the loss of cholinergic neurons and increased vesicular glutamate transporter 1 protein (VGLUT1) and glutamate decarboxylase (GAD) levels in the hippocampal tissue of rats with Aβ-induced AD (114). Therefore, Age may have great potential for the prevention and treatment of cardiogenic dementia.

*Salvia miltiorrhiza* (Danshen), as a classic traditional herbal medicine, has been widely used to treat heart disease because of its well-established pharmacological efficacies of the herb and its active components, including anti-atherosclerosis, anticardiac hypertrophy, anti-myocardial ischemia, anti-arrhythmia, endothelial cell damage repair, and improvement of coronary artery blood circulation (115). Importantly, *Salvia miltiorrhiza* and its active components have recently been shown to play a role in improving AD. Preclinical studies have indicated that the active ingredient tanshinone IIA in *Salvia miltiorrhiza* has excellent anti-inflammatory, antioxidant stress, and anti-apoptotic effects, which can significantly improve the learning and memory deficits of APP/PS1 transgenic AD mouse models (116). In addition, Tanshinone IIA can improve AD by upregulating the expression of Aβ-degrading enzymes such as insulin-degrading enzyme (IDE) and neplysine (NEP) (117). These results suggest that *Salvia miltiorrhiza* may be a potential therapeutic drug for cardiogenic dementia.

Ginseng is another traditional herbal medicine that has been used for thousands of years. Ginseng and its main active ingredient, ginsenosides, have been used to treat cardiac hypertrophy and HF because of their vasodilatory, antioxidant, anti-inflammatory, and anti-fibrotic properties (118, 119). Preclinical studies have shown that ginseng and ginsenosides improve cardiac function and reduce infarct size in rats with myocardial ischemia/reperfusion injury (MIRI), which is mainly facilitated by their inhibition of oxidative stress, inflammation, and fibrosis (120, 121). These are the prerequisites for ensuring the cardiac output of patients with cardiogenic dementia. In addition to their ability to improve the cognitive function of AD mice via reduction of Aβ deposition and tau hyperphosphorylation in the brain tissue, ginseng and ginsenosides also have excellent ability in improving CBF (122). It has been reported that ginsenosides significantly increase CBF in mice with bilateral common carotid artery ligation, which may be related to its vasodilatory effect (123). These findings indicate that ginseng and ginsenosides may serve as candidate drugs for cardiogenic dementia.

Our previous studies showed that Shuxuening injection, a *Ginkgo biloba* extract preparation, protects the cardiac function of MIRI mice via modulation of the TWEAK-Fn14 axis and improves cognition and motor deficits induced by cerebral ischemia/reperfusion injury (CIRI) by regulating hippocampal BDNF-mediated neurotrophin/Trk signaling (124–126). Ginkgo biloba extract (EGb 761®) is currently recommended by multiple guidelines for the treatment of mild cognitive impairment with or without cerebrovascular disease because of its known neuroprotective effects and cerebrovascular benefits (127). This indicates the therapeutic potential of Ginkgo biloba extract for cardiogenic dementia.

Qishen Yiqi pill (QSYQ), a Chinese compound medicine, has been reported to enhance cardiac output and CBF in patients with heart disease. A meta-analysis of 895 patients with HFpEF revealed that QSYQ improved diastolic function and successfully increased the rate of cardiac function recovery in patients with HFpEF (128). In addition, preclinical studies have reported that QSYQ increased CBF in mice with stroke (129). In our study, we found that QSYQ promoted the recovery of motor memory loss in CIRI rats by modulating ICAM-1-mediated neuroinflammation (130). This implies that QSYQ could be an effective medication for the treatment of cardiogenic dementia. However, additional validations are warranted.

## 5 Perspectives and conclusion

Cardiogenic dementia is becoming a prevalent condition that severely affects the diet, exercise, and mental status of patients and even polypharmacy, resulting in a poor quality of life and also imposing a huge economic burden on families and society. Early detection of cardiogenic dementia is necessary and should be conducted via enhanced neuropsychological tests, biomarker detection, and neuroimaging. Future research should further explore the complex relationship between the brain and heart, both at organ and systemic levels, to reveal the underlying mechanisms regulating the heart–brain interaction. Concerted efforts by a multidisciplinary team, which comprises cardiologists, neurologists, and basic and bioinformatic scientists, are essential to accomplish this goal. This will promote the development of effective management approaches for complications resulting from heart disease. More research is needed to determine the role of exosomes released by damaged cardiomyocytes in the occurrence of cognitive impairment. There is an urgent need to develop novel treatments for the cognitive dysfunction of patients with heart disease to reduce the incidence of cardiogenic dementia and improve the quality of life of patients. To this end, TCM-inspired or TCM-derived component-based and multitarget medicine may offer a fresh starting point. This study provides a reference for designing drugs for the clinical prevention and treatment of cardiogenic dementia.

## References

[1] Virani SS Alonso A Aparicio HJ Benjamin EJ Bittencourt MS Callaway CW Heart disease and stroke statistics-2021 update: a report from the American Heart Association. Circulation. (2021) 143(8):e254–743. 10.1161/cir.0000000000000950 33501848 PMC13036842

[2] Dridi H Liu Y Reiken S Liu X Argyrousi EK Yuan Q Heart failure-induced cognitive dysfunction is mediated by intracellular Ca(2+) leak through ryanodine receptor type 2. Nat Neurosci. (2023) 26(8):1365–78. 10.1038/s41593-023-01377-6 37429912 PMC10400432

[3] Havakuk O King KS Grazette L Yoon AJ Fong M Bregman N Heart failure-induced brain injury. J Am Coll Cardiol. (2017) 69(12):1609–16. 10.1016/j.jacc.2017.01.022 28335844

[4] Cardiogenic dementia. Lancet. (1977) 1(8001):27–8. 10.1016/S0140-6736(77)91660-9 63661

[5] Cardiogenic dementia. Lancet. (1981) 2(8256):1171. 10.1016/S0140-6736(81)90620-6 6118609

[6] Kisler K Nelson AR Montagne A Zlokovic BV. Cerebral blood flow regulation and neurovascular dysfunction in Alzheimer disease. Nat Rev Neurosci. (2017) 18(7):419–34. 10.1038/nrn.2017.48 28515434 PMC5759779

[7] Aryal R Patabendige A. Blood–brain barrier disruption in atrial fibrillation: a potential contributor to the increased risk of dementia and worsening of stroke outcomes? Open Biol. (2021) 11(4):200396. 10.1098/rsob.200396 33878948 PMC8059575

[8] Sposato LA Chaturvedi S. Atrial fibrillation detected after stroke and transient ischemic attack: a novel clinical concept challenging current views. Stroke. (2022) 53(3):e94–e103. 10.1161/strokeaha.121.034777 34986652

[9] Rydén L Zettergren A Seidu NM Guo X Kern S Blennow K Atrial fibrillation increases the risk of dementia amongst older adults even in the absence of stroke. J Intern Med. (2019) 286(1):101–10. 10.1111/joim.12902 30895641

[10] Nishtala A Piers RJ Himali JJ Beiser AS Davis-Plourde KL Saczynski JS Atrial fibrillation and cognitive decline in the Framingham heart study. Heart Rhythm. (2018) 15(2):166–72. 10.1016/j.hrthm.2017.09.036 28943482 PMC5881912

[11] Vella D Monteleone A Musotto G Bosi GM Burriesci G. Effect of the alterations in contractility and morphology produced by atrial fibrillation on the thrombosis potential of the left atrial appendage. Front Bioeng Biotechnol. (2021) 9:586041. 10.3389/fbioe.2021.586041 33718333 PMC7952649

[12] McFadyen J Peter K. Forget about thrombosis: platelets and Alzheimer’s disease, yet another sticky situation. Sci Signal. (2016) 9(429):fs9. 10.1126/scisignal.aaf8702 27221708

[13] Diener HC Hart RG Koudstaal PJ Lane DA Lip GYH. Atrial fibrillation and cognitive function: JJACC Review Topic of the Week. J Am Coll Cardiol. (2019) 73(5):612–9. 10.1016/j.jacc.2018.10.077 30732716

[14] Gardarsdottir M Sigurdsson S Aspelund T Rokita H Launer LJ Gudnason V Atrial fibrillation is associated with decreased total cerebral blood flow and brain perfusion. Europace. (2018) 20(8):1252–8. 10.1093/europace/eux220 29016776 PMC6075509

[15] Anselmino M Scarsoglio S Saglietto A Gaita F Ridolfi L. Transient cerebral hypoperfusion and hypertensive events during atrial fibrillation: a plausible mechanism for cognitive impairment. Sci Rep. (2016) 6:28635. 10.1038/srep28635 27334559 PMC4917883

[16] Bunch TJ. Atrial fibrillation and dementia. Circulation. (2020) 142(7):618–20. 10.1161/circulationaha.120.045866 32804567

[17] Koh YH Lew LZW Franke KB Elliott AD Lau DH Thiyagarajah A Predictive role of atrial fibrillation in cognitive decline: a systematic review and meta-analysis of 2.8 million individuals. Europace. (2022) 24(8):1229–39. 10.1093/europace/euac003 35061884 PMC9435641

[18] Xie W Zheng F Yan L Zhong B. Cognitive decline before and after incident coronary events. J Am Coll Cardiol. (2019) 73(24):3041–50. 10.1016/j.jacc.2019.04.019 31221251

[19] Anazodo UC Shoemaker JK Suskin N Ssali T Wang DJ St Lawrence KS. Impaired cerebrovascular function in coronary artery disease patients and recovery following cardiac rehabilitation. Front Aging Neurosci. (2015) 7:224. 10.3389/fnagi.2015.00224 26779011 PMC4700211

[20] Bélanger JC Bouchard V Le Blanc J Starnino L Welman M Chabot-Blanchet M Brain-derived neurotrophic factor mitigates the association between platelet dysfunction and cognitive impairment. Front Cardiovasc Med. (2021) 8:739045. 10.3389/fcvm.2021.739045 34557534 PMC8452906

[21] Ng TKS Coughlan C Heyn PC Tagawa A Carollo JJ Kua EH Increased plasma brain-derived neurotrophic factor (BDNF) as a potential biomarker for and compensatory mechanism in mild cognitive impairment: a case–control study. Aging (Albany NY). (2021) 13(19):22666–89. 10.18632/aging.203598 34607976 PMC8544315

[22] Liu S Fan M Xu JX Yang LJ Qi CC Xia QR Exosomes derived from bone-marrow mesenchymal stem cells alleviate cognitive decline in AD-like mice by improving BDNF-related neuropathology. J Neuroinflammation. (2022) 19(1):35. 10.1186/s12974-022-02393-2 35130907 PMC8822863

[23] Ishihara K Izawa KP Kitamura M Shimogai T Kanejima Y Morisawa T Serum concentration of dihomo-γ-linolenic acid is associated with cognitive function and mild cognitive impairment in coronary artery disease patients. Prostaglandins Leukot Essent Fatty Acids. (2020) 158:102038. 10.1016/j.plefa.2019.102038 31767440

[24] Gharacholou SM Reid KJ Arnold SV Spertus J Rich MW Pellikka PA Cognitive impairment and outcomes in older adult survivors of acute myocardial infarction: findings from the translational research investigating underlying disparities in acute myocardial infarction patients’ health status registry. Am Heart J. (2011) 162(5):860–869.e1. 10.1016/j.ahj.2011.08.005 22093202 PMC3410733

[25] Ikram MA van Oijen M de Jong FJ Kors JA Koudstaal PJ Hofman A Unrecognized myocardial infarction in relation to risk of dementia and cerebral small vessel disease. Stroke. (2008) 39(5):1421–6. 10.1161/strokeaha.107.501106 18323497

[26] Di Bella G Aquaro GD Bogaert J Piaggi P Micari A Pizzino F Non-transmural myocardial infarction associated with regional contractile function is an independent predictor of positive outcome: an integrated approach to myocardial viability. J Cardiovasc Magn Reson. (2021) 23(1):121. 10.1186/s12968-021-00818-0 34719402 PMC8559354

[27] Marving J Høilund-Carlsen PF Chraemmer-Jørgensen B Gadsbøll N. Are right and left ventricular ejection fractions equal? Ejection fractions in normal subjects and in patients with first acute myocardial infarction. Circulation. (1985) 72(3):502–14. 10.1161/01.cir.72.3.502 4017205

[28] Menon V Slater JN White HD Sleeper LA Cocke T Hochman JS. Acute myocardial infarction complicated by systemic hypoperfusion without hypotension: report of the SHOCK trial registry. Am J Med. (2000) 108(5):374–80. 10.1016/s0002-9343(00)00310-7 10759093

[29] Thorp EB Flanagan ME Popko B DeBerge M. Resolving inflammatory links between myocardial infarction and vascular dementia. Semin Immunol. (2022) 59:101600. 10.1016/j.smim.2022.101600 35227567 PMC10234261

[30] Borchert T Hess A Lukačević M Ross TL Bengel FM Thackeray JT. Angiotensin-converting enzyme inhibitor treatment early after myocardial infarction attenuates acute cardiac and neuroinflammation without effect on chronic neuroinflammation. Eur J Nucl Med Mol Imaging. (2020) 47(7):1757–68. 10.1007/s00259-020-04736-8 32125488 PMC7248052

[31] Thackeray JT Hupe HC Wang Y Bankstahl JP Berding G Ross TL Myocardial inflammation predicts remodeling and neuroinflammation after myocardial infarction. J Am Coll Cardiol. (2018) 71(3):263–75. 10.1016/j.jacc.2017.11.024 29348018

[32] Sundbøll J Horváth-Puhó E Adelborg K Schmidt M Pedersen L Bøtker HE Higher risk of vascular dementia in myocardial infarction survivors. Circulation. (2018) 137(6):567–77. 10.1161/circulationaha.117.029127 29025764

[33] Wolters FJ Segufa RA Darweesh SKL Bos D Ikram MA Sabayan B Coronary heart disease, heart failure, and the risk of dementia: a systematic review and meta-analysis. Alzheimer’s & Dement. (2018) 14(11):1493–504. 10.1016/j.jalz.2018.01.007 29494808

[34] Goh FQ Kong WKF Wong RCC Chong YF Chew NWS Yeo TC Cognitive impairment in heart failure-a review. Biology (Basel). (2022) 11(2):179. 10.3390/biology11020179 35205045 PMC8869585

[35] Myers SJ Jiménez-Ruiz A Sposato LA Whitehead SN. Atrial cardiopathy and cognitive impairment. Front Aging Neurosci. (2022) 14:914360. 10.3389/fnagi.2022.914360 35942230 PMC9355976

[36] Redwine LS Hong S Kohn J Martinez C Hurwitz BE Pung MA Systemic inflammation and cognitive decrements in patients with stage B heart failure. Psychosom Med. (2022) 84(2):133–40. 10.1097/psy.0000000000001033 34654027

[37] Faulkner KM Dickson VV Fletcher J Katz SD Chang PP Gottesman RF Factors associated with cognitive impairment in heart failure with preserved ejection fraction. J Cardiovasc Nurs. (2022) 37(1):17–30. 10.1097/jcn.0000000000000711 32649377 PMC9069246

[38] de Bruijn RF Portegies ML Leening MJ Bos MJ Hofman A van der Lugt A Subclinical cardiac dysfunction increases the risk of stroke and dementia: the Rotterdam study. Neurology. (2015) 84(8):833–40. 10.1212/wnl.0000000000001289 25632093

[39] Adelborg K Horváth-Puhó E Ording A Pedersen L Sørensen HT Henderson VW. Heart failure and risk of dementia: a Danish nationwide population-based cohort study. Eur J Heart Fail. (2017) 19(2):253–60. 10.1002/ejhf.631 27612177 PMC5522185

[40] Rodriguez CJ Bartz TM Longstreth WT Jr Kizer JR Barasch E Lloyd-Jones DM Association of annular calcification and aortic valve sclerosis with brain findings on magnetic resonance imaging in community dwelling older adults: the cardiovascular health study. J Am Coll Cardiol. (2011) 57(21):2172–80. 10.1016/j.jacc.2011.01.034 21596233 PMC4104125

[41] Doenst T Pytel G Schrepper A Amorim P Färber G Shingu Y Decreased rates of substrate oxidation ex vivo predict the onset of heart failure and contractile dysfunction in rats with pressure overload. Cardiovasc Res. (2010) 86(3):461–70. 10.1093/cvr/cvp414 20035032

[42] Sung SH Lee CW Wang PN Lee HY Chen CH Chung CP. Cognitive functions and jugular venous reflux in severe mitral regurgitation: a pilot study. PLoS One. (2019) 14(2):e0207832. 10.1371/journal.pone.0207832 30794544 PMC6386300

[43] Koike A Itoh H Oohara R Hoshimoto M Tajima A Aizawa T Cerebral oxygenation during exercise in cardiac patients. Chest. (2004) 125(1):182–90. 10.1378/chest.125.1.182 14718439

[44] Junejo RT Lip GYH Fisher JP. Cerebrovascular dysfunction in atrial fibrillation. Front Physiol. (2020) 11:1066. 10.3389/fphys.2020.01066 33013456 PMC7509200

[45] Ovsenik A Podbregar M Fabjan A. Cerebral blood flow impairment and cognitive decline in heart failure. Brain Behav. (2021) 11(6):e02176. 10.1002/brb3.2176 33991075 PMC8213942

[46] Ainslie PN Duffin J. Integration of cerebrovascular CO2 reactivity and chemoreflex control of breathing: mechanisms of regulation, measurement, and interpretation. Am J Physiol Regul Integr Comp Physiol. (2009) 296(5):R1473–1495. 10.1152/ajpregu.91008.2008 19211719

[47] Xie A Skatrud JB Khayat R Dempsey JA Morgan B Russell D. Cerebrovascular response to carbon dioxide in patients with congestive heart failure. Am J Respir Crit Care Med. (2005) 172(3):371–8. 10.1164/rccm.200406-807OC 15901613

[48] Georgiadis D Sievert M Cencetti S Uhlmann F Krivokuca M Zierz S Cerebrovascular reactivity is impaired in patients with cardiac failure. Eur Heart J. (2000) 21(5):407–13. 10.1053/euhj.1999.1742 10666355

[49] Aires A Andrade A Azevedo E. Neurovascular coupling impairment in heart failure with reduction ejection fraction. Brain Sci. (2020) 10(10):714. 10.3390/brainsci10100714 33036338 PMC7601077

[50] Riegger AJ. Role of vasopressin in experimental heart failure. Eur Heart J. (1988) 9(Suppl H):7–10. 10.1093/eurheartj/9.suppl_h.7 3049096

[51] Junejo RT Braz ID Lucas SJ van Lieshout JJ Phillips AA Lip GY Neurovascular coupling and cerebral autoregulation in atrial fibrillation. J Cereb Blood Flow Metab. (2020) 40(8):1647–57. 10.1177/0271678X19870770 31426699 PMC7370373

[52] Graff-Radford J Madhavan M Vemuri P Rabinstein AA Cha RH Mielke MM Atrial fibrillation, cognitive impairment, and neuroimaging. Alzheimer’s Dement. (2016) 12(4):391–8. 10.1016/j.jalz.2015.08.164 26607820 PMC4841716

[53] Butterfield DA Halliwell B. Oxidative stress, dysfunctional glucose metabolism and Alzheimer disease. Nat Rev Neurosci. (2019) 20(3):148–60. 10.1038/s41583-019-0132-6 30737462 PMC9382875

[54] Toledo C Andrade DC Díaz HS Inestrosa NC Del Rio R. Neurocognitive disorders in heart failure: novel pathophysiological mechanisms underpinning memory loss and learning impairment. Mol Neurobiol. (2019) 56(12):8035–51. 10.1007/s12035-019-01655-0 31165973

[55] Zhao Y Zhang J Zheng Y Zhang Y Zhang XJ Wang H NAD(+) improves cognitive function and reduces neuroinflammation by ameliorating mitochondrial damage and decreasing ROS production in chronic cerebral hypoperfusion models through Sirt1/PGC-1*α* pathway. J Neuroinflammation. (2021) 18(1):207. 10.1186/s12974-021-02250-8 34530866 PMC8444613

[56] Yan N Xu Z Qu C Zhang J. Dimethyl fumarate improves cognitive deficits in chronic cerebral hypoperfusion rats by alleviating inflammation, oxidative stress, and ferroptosis via NRF2/ARE/NF-κB signal pathway. Int Immunopharmacol. (2021) 98:107844. 10.1016/j.intimp.2021.107844 34153667

[57] Camacho-Castillo L Phillips-Farfán BV Rosas-Mendoza G Baires-López A Toral-Ríos D Campos-Peña V Increased oxidative stress contributes to enhance brain amyloidogenesis and blunts energy metabolism in sucrose-fed rat: effect of AMPK activation. Sci Rep. (2021) 11(1):19547. 10.1038/s41598-021-98983-w 34599229 PMC8486781

[58] Emoto MC Sato-Akaba H Hamaue N Kawanishi K Koshino H Shimohama S Early detection of redox imbalance in the APPswe/PS1dE9 mouse model of Alzheimer’s disease by in vivo electron paramagnetic resonance imaging. Free Radic Biol Med. (2021) 172:9–18. 10.1016/j.freeradbiomed.2021.05.035 34058322

[59] Khan A Park TJ Ikram M Ahmad S Ahmad R Jo MG Antioxidative and anti-inflammatory effects of kojic acid in aβ-induced mouse model of Alzheimer’s disease. Mol Neurobiol. (2021) 58(10):5127–40. 10.1007/s12035-021-02460-4 34255249

[60] Vidyanti AN Hsieh JY Lin KJ. Role of HMGB1 in an animal model of vascular cognitive impairment induced by chronic cerebral hypoperfusion. Int J Mol Sci. (2020) 21(6):2176. 10.3390/ijms21062176 32245271 PMC7139598

[61] Imen T Salma M Khouloud C Habib GM Kaouthar B Nejia T IL-1β gene polymorphism and serum levels in a Tunisian population with acute heart failure. Biomark Med. (2017) 11(12):1069–76. 10.2217/bmm-2017-0179 29182005

[62] Shaftel SS Griffin WS O'Banion MK. The role of interleukin-1 in neuroinflammation and Alzheimer disease: an evolving perspective. J Neuroinflammation. (2008) 5:7. 10.1186/1742-2094-5-7 18302763 PMC2335091

[63] Gonzalez P Machado I Vilcaes A Caruso C Roth GA Schiöth H Molecular mechanisms involved in interleukin 1-beta (IL-1β)-induced memory impairment. Modulation by alpha-melanocyte-stimulating hormone (α-MSH). Brain Behav Immun. (2013) 34:141–50. 10.1016/j.bbi.2013.08.007 23968970

[64] Brás JP Bravo J Freitas J Barbosa MA Santos SG Summavielle T TNF-alpha-induced microglia activation requires miR-342: impact on NF-kB signaling and neurotoxicity. Cell Death Dis. (2020) 11(6):415. 10.1038/s41419-020-2626-6 32488063 PMC7265562

[65] Toyama K Koibuchi N Uekawa K Hasegawa Y Kataoka K Katayama T Apoptosis signal-regulating kinase 1 is a novel target molecule for cognitive impairment induced by chronic cerebral hypoperfusion. Arterioscler Thromb Vasc Biol. (2014) 34(3):616–25. 10.1161/atvbaha.113.302440 24371084

[66] Hou X Liang X Chen JF Zheng J. Ecto-5'-nucleotidase (CD73) is involved in chronic cerebral hypoperfusion-induced white matter lesions and cognitive impairment by regulating glial cell activation and pro-inflammatory cytokines. Neuroscience. (2015) 297:118–26. 10.1016/j.neuroscience.2015.03.033 25805696

[67] Salvadores N Searcy JL Holland PR Horsburgh K. Chronic cerebral hypoperfusion alters amyloid-β peptide pools leading to cerebral amyloid angiopathy, microinfarcts and haemorrhages in tg-SwDI mice. Clin Sci (Lond). (2017) 131(16):2109–23. 10.1042/cs20170962 28667120

[68] Bannai T Mano T Chen X Ohtomo G Ohtomo R Tsuchida T Chronic cerebral hypoperfusion shifts the equilibrium of amyloid β oligomers to aggregation-prone species with higher molecular weight. Sci Rep. (2019) 9(1):2827. 10.1038/s41598-019-39494-7 30808940 PMC6391466

[69] Galenko O Jacobs V Knight S Bride D Cutler MJ Muhlestein JB Circulating levels of biomarkers of cerebral injury in patients with atrial fibrillation. Am J Cardiol. (2019) 124(11):1697–700. 10.1016/j.amjcard.2019.08.027 31575426

[70] Althammer F Ferreira-Neto HC Rubaharan M Roy RK Patel AA Murphy A Three-dimensional morphometric analysis reveals time-dependent structural changes in microglia and astrocytes in the central amygdala and hypothalamic paraventricular nucleus of heart failure rats. J Neuroinflammation. (2020) 17(1):221. 10.1186/s12974-020-01892-4 32703230 PMC7379770

[71] Han B Jiang W Liu H Wang J Zheng K Cui P Upregulation of neuronal PGC-1α ameliorates cognitive impairment induced by chronic cerebral hypoperfusion. Theranostics. (2020) 10(6):2832–48. 10.7150/thno.37119 32194838 PMC7052889

[72] Hansen DV Hanson JE Sheng M. Microglia in Alzheimer’s disease. J Cell Biol. (2018) 217(2):459–72. 10.1083/jcb.201709069 29196460 PMC5800817

[73] Maphis N Xu G Kokiko-Cochran ON Jiang S Cardona A Ransohoff RM Reactive microglia drive tau pathology and contribute to the spreading of pathological tau in the brain. Brain. (2015) 138(Pt 6):1738–55. 10.1093/brain/awv081 25833819 PMC4542622

[74] Bascuñana P Hess A Borchert T Wang Y Wollert KC Bengel FM ^11^C-methionine PET identifies astroglia involvement in heart–brain inflammation networking after acute myocardial infarction. J Nucl Med. (2020) 61(7):977–80. 10.2967/jnumed.119.236885 31806766 PMC7383078

[75] Sabayan B van Buchem MA Sigurdsson S Zhang Q Meirelles O Harris TB Cardiac and carotid markers link with accelerated brain atrophy: the AGES-Reykjavik study (age, gene/environment susceptibility-Reykjavik). Arterioscler Thromb Vasc Biol. (2016) 36(11):2246–51. 10.1161/atvbaha.116.308018 27609370 PMC5310810

[76] Wong SM Jansen JFA Zhang CE Hoff EI Staals J van Oostenbrugge RJ Blood–brain barrier impairment and hypoperfusion are linked in cerebral small vessel disease. Neurology. (2019) 92(15):e1669–77. 10.1212/wnl.0000000000007263 30867275

[77] Sun Z Gao C Gao D Sun R Li W Wang F Reduction in pericyte coverage leads to blood-brain barrier dysfunction via endothelial transcytosis following chronic cerebral hypoperfusion. Fluids Barriers CNS. (2021) 18(1):21. 10.1186/s12987-021-00255-2 33952281 PMC8101037

[78] Liu Q Radwanski R Babadjouni R Patel A Hodis DM Baumbacher P Experimental chronic cerebral hypoperfusion results in decreased pericyte coverage and increased blood-brain barrier permeability in the corpus callosum. J Cereb Blood Flow Metab. (2019) 39(2):240–50. 10.1177/0271678X17743670 29192539 PMC6365610

[79] Yang H Wang W Jia L Qin W Hou T Wu Q The effect of chronic cerebral hypoperfusion on blood-brain barrier permeability in a transgenic Alzheimer’s disease mouse model (PS1V97l). J Alzheimers Dis. (2020) 74(1):261–75. 10.3233/jad-191045 32007956

[80] Toledo C Lucero C Andrade DC Díaz HS Schwarz KG Pereyra KV Cognitive impairment in heart failure is associated with altered wnt signaling in the hippocampus. Aging. (2019) 11(16):5924–42. 10.18632/aging.102150 31447429 PMC6738419

[81] Gao S Li L Li L Ni J Guo R Mao J Effects of the combination of tanshinone IIA and puerarin on cardiac function and inflammatory response in myocardial ischemia mice. J Mol Cell Cardiol. (2019) 137:59–70. 10.1016/j.yjmcc.2019.09.012 31629735

[82] Wang X Guo Z Ding Z Mehta JL. Inflammation, autophagy, and apoptosis after myocardial infarction. J Am Heart Assoc. (2018) 7(9):e008024. 10.1161/jaha.117.008024 29680826 PMC6015297

[83] Yang J Ran M Li H Lin Y Ma K Yang Y New insight into neurological degeneration: inflammatory cytokines and blood-brain barrier. Front Mol Neurosci. (2022) 15:1013933. 10.3389/fnmol.2022.1013933 36353359 PMC9637688

[84] Fard M T Stough C. A review and hypothesized model of the mechanisms that underpin the relationship between inflammation and cognition in the elderly. Front Aging Neurosci. (2019) 11:56. 10.3389/fnagi.2019.00056 30930767 PMC6425084

[85] Rundqvist B Elam M Bergmann-Sverrisdottir Y Eisenhofer G Friberg P. Increased cardiac adrenergic drive precedes generalized sympathetic activation in human heart failure. Circulation. (1997) 95(1):169–75. 10.1161/01.cir.95.1.169 8994433

[86] Parent MB Ferreira-Neto HC Kruemmel AR Althammer F Patel AA Keo S Heart failure impairs mood and memory in male rats and down-regulates the expression of numerous genes important for synaptic plasticity in related brain regions. Behav Brain Res. (2021) 414:113452. 10.1016/j.bbr.2021.113452 34274373 PMC9488982

[87] de Lucia C Femminella GD Gambino G Pagano G Allocca E Rengo C Adrenal adrenoceptors in heart failure. Front Physiol. (2014) 5:246. 10.3389/fphys.2014.00246 25071591 PMC4084669

[88] Taurin S Sandbo N Qin Y Browning D Dulin NO. Phosphorylation of beta-catenin by cyclic AMP-dependent protein kinase. J Biol Chem. (2006) 281(15):9971–6. 10.1074/jbc.M508778200 16476742

[89] Verheyen EM Gottardi CJ. Regulation of Wnt/beta-catenin signaling by protein kinases. Dev Dyn. (2010) 239(1):34–44. 10.1002/dvdy.22019 19623618 PMC3173947

[90] Inestrosa NC Arenas E. Emerging roles of Wnts in the adult nervous system. Nat Rev Neurosci. (2010) 11(2):77–86. 10.1038/nrn2755 20010950

[91] Gruhn N Larsen FS Boesgaard S Knudsen GM Mortensen SA Thomsen G Cerebral blood flow in patients with chronic heart failure before and after heart transplantation. Stroke. (2001) 32(11):2530–3. 10.1161/hs1101.098360 11692012

[92] Noureddine FY Altara R. Impact of the renin-angiotensin system on the endothelium in vascular dementia: unresolved issues and future perspectives. Int J Mol Sci. (2020) 21(12):4268. 10.3390/ijms21124268 32560034 PMC7349348

[93] Zheng X Hermann DM Bähr M Doeppner TR. The role of small extracellular vesicles in cerebral and myocardial ischemia-molecular signals, treatment targets, and future clinical translation. Stem Cells. (2021) 39(4):403–13. 10.1002/stem.3329 33432732

[94] Rodriguez JA Orbe J Saenz-Pipaon G Abizanda G Gebara N Radulescu F Selective increase of cardiomyocyte derived extracellular vesicles after experimental myocardial infarction and functional effects on the endothelium. Thromb Res. (2018) 170:1–9. 10.1016/j.thromres.2018.07.030 30081387

[95] Biemmi V Milano G Ciullo A Cervio E Burrello J Dei Cas M Inflammatory extracellular vesicles prompt heart dysfunction via TRL4-dependent NF-κB activation. Theranostics. (2020) 10(6):2773–90. 10.7150/thno.39072 32194834 PMC7052909

[96] Matsumoto J Stewart T Sheng L Li N Bullock K Song N Transmission of α-synuclein-containing erythrocyte-derived extracellular vesicles across the blood-brain barrier via adsorptive mediated transcytosis: another mechanism for initiation and progression of Parkinson’s disease? Acta Neuropathol Commun. (2017) 5(1):71. 10.1186/s40478-017-0470-4 28903781 PMC5598000

[97] Li JJ Wang B Kodali MC Chen C Kim E Patters BJ In vivo evidence for the contribution of peripheral circulating inflammatory exosomes to neuroinflammation. J Neuroinflammation. (2018) 15(1):8. 10.1186/s12974-017-1038-8 29310666 PMC5759808

[98] Gelosa P Castiglioni L Rzemieniec J Muluhie M Camera M Sironi L. Cerebral derailment after myocardial infarct: mechanisms and effects of the signaling from the ischemic heart to brain. J Mol Med (Berl). (2022) 100(1):23–41. 10.1007/s00109-021-02154-3 34674004 PMC8724191

[99] Sun LL Duan MJ Ma JC Xu L Mao M Biddyut D Myocardial infarction-induced hippocampal microtubule damage by cardiac originating microRNA-1 in mice. J Mol Cell Cardiol. (2018) 120:12–27. 10.1016/j.yjmcc.2018.05.009 29775643

[100] Duan MJ Yan ML Wang Q Mao M Su D Sun LL Overexpression of miR-1 in the heart attenuates hippocampal synaptic vesicle exocytosis by the posttranscriptional regulation of SNAP-25 through the transportation of exosomes. Cell Commun Signal. (2018) 16(1):91. 10.1186/s12964-018-0303-5 30497498 PMC6267908

[101] Chi F Feng L Li Y Zhao S Yuan W Jiang Y MiR-30b-5p promotes myocardial cell apoptosis in rats with myocardial infarction through regulating Wnt/β-catenin signaling pathway. Minerva Med. (2023) 114(4):476–84. 10.23736/s0026-4806.20.06565-9 32255311

[102] Zhang F Cheng N Han Y Zhang C Zhang H. miRNA expression profiling uncovers a role of miR-139-5p in regulating the calcification of human aortic valve interstitial cells. Front Genet. (2021) 12:722564. 10.3389/fgene.2021.722564 34745206 PMC8569802

[103] Kim MY Jung M Noh Y Shin S Hong CH Lee S Impact of statin use on dementia incidence in elderly men and women with ischemic heart disease. Biomedicines. (2020) 8(2):30. 10.3390/biomedicines8020030 32050497 PMC7168191

[104] Husain I Akhtar M Vohora D Abdin MZ Islamuddin M Akhtar MJ Rosuvastatin attenuates high-salt and cholesterol diet induced neuroinflammation and cognitive impairment via preventing nuclear factor kappaB pathway. Neurochem Res. (2017) 42(8):2404–16. 10.1007/s11064-017-2264-2 28417263

[105] Swiger KJ Manalac RJ Blumenthal RS Blaha MJ Martin SS. Statins and cognition: a systematic review and meta-analysis of short- and long-term cognitive effects. Mayo Clin Proc. (2013) 88(11):1213–21. 10.1016/j.mayocp.2013.07.013 24095248

[106] Lee SR Choi EK. Comparing warfarin and 4 direct oral anticoagulants for the risk of dementia in patients with atrial fibrillation. Stroke. (2021) 52(11):3459–68. 10.1161/strokeaha.120.033338 34496627

[107] Cadogan SL Powell E Wing K Wong AY Smeeth L. Anticoagulant prescribing for atrial fibrillation and risk of incident dementia. Heart. (2021) 107(23):1898–904. 10.1136/heartjnl-2021-319672 34645643 PMC8600601

[108] Grossmann K. Direct oral anticoagulants (DOACs) for therapeutic targeting of thrombin, a key mediator of cerebrovascular and neuronal dysfunction in Alzheimer’s disease. Biomedicines. (2022) 10(8):1890. 10.3390/biomedicines10081890 36009437 PMC9405823

[109] Kim MY Noh Y Son SJ Shin S Paik HY Lee S Effect of cilostazol on incident dementia in elderly men and women with ischemic heart disease. J Alzheimer’s Dis. (2018) 63(2):635–44. 10.3233/jad-170895 29660935

[110] Ding MR Qu YJ Hu B An HM. Signal pathways in the treatment of Alzheimer’s disease with traditional Chinese medicine. Biomed Pharmacother. (2022) 152:113208. 10.1016/j.biopha.2022.113208 35660246

[111] Luo JF Dong Y Chen JY Lu JH. The effect and underlying mechanisms of garlic extract against cognitive impairment and Alzheimer’s disease: a systematic review and meta-analysis of experimental animal studies. J Ethnopharmacol. (2021) 280:114423. 10.1016/j.jep.2021.114423 34273446

[112] Lindstedt S Wlosinska M Nilsson AC Hlebowicz J Fakhro M Sheikh R. Successful improved peripheral tissue perfusion was seen in patients with atherosclerosis after 12 months of treatment with aged garlic extract. Int Wound J. (2021) 18(5):681–91. 10.1111/iwj.13570 33590955 PMC8450802

[113] Morihara N Sumioka I Moriguchi T Uda N Kyo E. Aged garlic extract enhances production of nitric oxide. Life Sci. (2002) 71(5):509–17. 10.1016/s0024-3205(02)01706-x 12052435

[114] Thorajak P Pannangrong W Welbat JU Chaijaroonkhanarak W Sripanidkulchai K Sripanidkulchai B. Effects of aged garlic extract on cholinergic, glutamatergic and GABAergic systems with regard to cognitive impairment in aβ-induced rats. Nutrients. (2017) 9(7):686. 10.3390/nu9070686 28671572 PMC5537801

[115] Guo R Li L Su J Li S Duncan SE Liu Z Pharmacological activity and mechanism of tanshinone IIA in related diseases. Drug Des Devel Ther. (2020) 14:4735–48. 10.2147/dddt.S266911 33192051 PMC7653026

[116] Peng X Chen L Wang Z He Y Ruganzu JB Guo H Tanshinone IIA regulates glycogen synthase kinase-3β-related signaling pathway and ameliorates memory impairment in APP/PS1 transgenic mice. Eur J Pharmacol. (2022) 918:174772. 10.1016/j.ejphar.2022.174772 35090935

[117] Liu XQ Hu T Wu GL Qiao LJ Cai YF Wang Q Tanshinone IIA, the key compound in *Salvia miltiorrhiza*, improves cognitive impairment by upregulating Aβ-degrading enzymes in APP/PS1 mice. Int J Biol Macromol. (2024) 254(Pt 2):127923. 10.1016/j.ijbiomac.2023.127923 37944734

[118] Li C Zhang X Li J Liang L Zeng J Wen M Ginsenoside Rb1 promotes the activation of PPAR*α* pathway via inhibiting FADD to ameliorate heart failure. Eur J Pharmacol. (2023) 947:175676. 10.1016/j.ejphar.2023.175676 37001580

[119] Ren B Feng J Yang N Guo Y Chen C Qin Q. Ginsenoside Rg3 attenuates angiotensin II-induced myocardial hypertrophy through repressing NLRP3 inflammasome and oxidative stress via modulating SIRT1/NF-κB pathway. Int Immunopharmacol. (2021) 98:107841. 10.1016/j.intimp.2021.107841 34153662

[120] Li L Wang Y Guo R Li S Ni J Gao S Ginsenoside Rg3-loaded, reactive oxygen species-responsive polymeric nanoparticles for alleviating myocardial ischemia-reperfusion injury. J Controlled Release. (2020) 317:259–72. 10.1016/j.jconrel.2019.11.032 PMC7384207 31783047

[121] Wang Q Fu W Yu X Xu H Sui D Wang Y. Ginsenoside Rg2 alleviates myocardial fibrosis by regulating TGF-β1/Smad signalling pathway. Pharm Biol. (2021) 59(1):106–13. 10.1080/13880209.2020.1867197 33535854 PMC8871615

[122] Huang X Li N Pu Y Zhang T Wang B. Neuroprotective effects of ginseng phytochemicals: recent perspectives. Molecules. (2019) 24(16):2939. 10.3390/molecules24162939 31416121 PMC6720911

[123] Han Y Li X Yang L Zhang D Li L Dong X Ginsenoside Rg1 attenuates cerebral ischemia–reperfusion injury due to inhibition of NOX2-mediated calcium homeostasis dysregulation in mice. J Ginseng Res. (2022) 46(4):515–25. 10.1016/j.jgr.2021.08.001 35818419 PMC9270650

[124] Li Z Wang H Xiao G Du H He S Feng Y Recovery of post-stroke cognitive and motor deficiencies by Shuxuening injection via regulating hippocampal BDNF-mediated neurotrophin/Trk signaling. Biomed Pharmacother. (2021) 141:111828. 10.1016/j.biopha.2021.111828 34146848

[125] Li Z Xiao G Lyu M Wang Y He S Du H Shuxuening injection facilitates neurofunctional recovery via down-regulation of G-CSF-mediated granulocyte adhesion and diapedesis pathway in a subacute stroke mouse model. Biomed Pharmacother. (2020) 127:110213. 10.1016/j.biopha.2020.110213 32417690

[126] Xiao G Lyu M Wang Y He S Liu X Ni J Ginkgo flavonol glycosides or ginkgolides tend to differentially protect myocardial or cerebral ischemia-reperfusion injury via regulation of TWEAK-Fn14 signaling in heart and brain. Front Pharmacol. (2019) 10:735. 10.3389/fphar.2019.00735 31333457 PMC6624656

[127] Kandiah N Chan YF Chen C Dasig D Dominguez J Han SH Strategies for the use of *Ginkgo biloba* extract, EGb 761(®), in the treatment and management of mild cognitive impairment in Asia: expert consensus. CNS Neurosci Ther. (2021) 27(2):149–62. 10.1111/cns.13536 33352000 PMC7816207

[128] Wang M Shan Y Wu C Cao P Sun W Han J Efficacy and safety of Qishen Yiqi dripping pill for heart failure with preserved ejection fraction: a systematic review and meta-analysis. Front Pharmacol. (2020) 11:626375. 10.3389/fphar.2020.626375 33633570 PMC7900630

[129] Ye Y Li Q Pan CS Yan L Sun K Wang XY Qishenyiqi inhibits tissue plasminogen activator-induced brain edema and hemorrhage after ischemic stroke in mice. Front Pharmacol. (2021) 12:759027. 10.3389/fphar.2021.759027 35095486 PMC8790519

[130] Liu X Xiao G Wang Y Shang T Li Z Wang H Qishen Yiqi dropping pill facilitates post-stroke recovery of motion and memory loss by modulating ICAM-1-mediated neuroinflammation. Biomed Pharmacother. (2022) 153:113325. 10.1016/j.biopha.2022.113325 35772377

